# Supplementary material for: Optimization of γ-Aminobutyric Acid Production in Brown Rice via Prolonged Seed Priming
Source: Plants (Basel). 2024 Dec 23;13(24):3594. doi: 10.3390/plants13243594 (PMC11677616; doi:10.3390/plants13243594)
Supplement: Supplementary file 1 [file plants-13-03594-s001.zip › plants-3369860-supplementary.pdf]

Table S1. Comparison of various brown rice processing methods.

	Advantage	Disadvantage (probable solution)
PLP	rapid GABA synthesis along with a series of nutrients<br>predictable germinability<br>storability<br>a possible standard for grain GABA content in variety test (without farming)	by products with oxidative stress (multi-germination stage, multi-products study to time the most nutritive stage)<br>early germinated proportion vulnerable to desiccation injury (using desiccation tolerant varieties; discarding sprouted grained before redrying)
Routine priming	hardly desiccation damage<br>long-time storage	grain broken during dehulling<br>GABA synthesis not rapid enough<br>unpredictable germinablity
no priming + long farming	high GABA content along with other nutrients	time consuming<br>leaching of pigments<br>unpredictable germinability

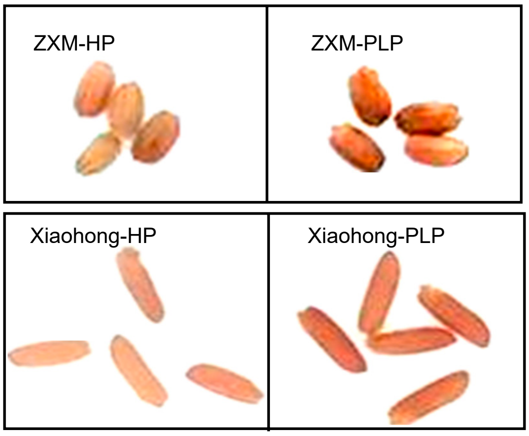

Figure S1. Impact of PLP on the bran color of ZXM and Xiaohong
